# Open debate and progress in ecology and evolution

**DOI:** 10.1002/ece3.5

**Published:** 2011-09

**Authors:** Allen J Moore


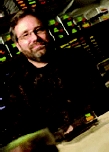


It is a fundamental obligation of scientists to publish their work, to make it accessible to a wide audience and to contribute this knowledge to the community. Yes, a “publish or perish” culture still lives, but this is in large part because researchers want to publish their results and know and respect the value of publishing. Research results that go unpublished are simply a hobby. Research that is published but not read or acted upon is a tragedy. Researchers know this, and in my experience they spend considerable time, thought and discussion over where they can publish their results. Where will their research be seen, and therefore cited most often? Which journal reaches the right audience? No one submits work for publication without expecting it to be ultimately accepted and published – and then to have influence over the field. Alongside the considerable work of the authors to produce high-quality research results and manuscripts, editors and reviewers work to ensure that their journal maintains its quality, impact and prestige.

This system works well, but there is a natural tension. Editors of excellent journals realise that they will end up rejecting well over two thirds of the papers that are submitted. Authors all expect their manuscripts to be in that top third but not all are. Furthermore, editors aren't sufficiently broad to be expert on every topic in their journal and we therefore invite opinion from knowledgeable reviewers. Reviewers also know the statistics, so when reading a paper they assess not only the quality of the science and writing, but also the suitability of the work for the journal for which they are reviewing. Editors pick reviewers who they believe to be expert and have the judgement to help them pick out the papers that belong in their journal.

There are a number of reasons a paper may not be accepted. There may be fatal flaws, such as a lack of sufficient controls, inappropriate analyses, pseudoreplication, etc. In my experience, this is relatively unusual. More typically, there are flaws that can be corrected. Often alternative analyses show the same thing, but they are requested by reviewers because statistics has moved on and there are newer, preferred methods. At first glance, the need for new analyses appears fatal, but isn't because the new analyses do not change the interpretations or conclusions. Another reason a paper is rejected is because it “just doesn't pass the quality threshold for our journal”. This is entirely subjective, but often reflects the sense that the work is confirmatory of a large body of already published papers, is taxon-specific, lacks sufficient sample size, or just isn't exciting. Often the real reason for rejection, whatever the detailed criticisms of the reviewers, is that the authors failed to communicate their ideas clearly, resulting in poorly argued arguments, or poorly explained methods or hypotheses, which lead reviewers to misunderstand what was being presented. This is the fault of the author, not the reviewer, but it is does frustrate authors because it can be fixed. And finally, papers are usually not accepted when they receive conflicting reviews. This may not be fair or desirable, but faced with maintaining a high rejection rate, what is an editor to do? Rejection is understandable from the editor's perspective; the reviewers were chosen to advise based on an expectation that the chosen reviewer was qualified, fair, and knowledgeable. In the case of a disagreement, the advantage naturally goes to the reviewer.

None of this would matter except for three things: first, we are wasting the time and valuable effort of the reviewer community if the original reviews were valid. Second, faced with rejecting most of the papers that are received, editors are unable to accept many excellent papers and thus waste the time of the authors and delay the publication of their work. And finally, if editors solicit two or three reviews, a single negative review is enough to get a paper rejected despite one or more positive reviews indicating a paper should be accepted for publication. The result of rejection is (one hopes) that the authors revise the paper. But this means tailoring it for another journal and yet another round of reviews. While the review process is important, and results in far superior research papers, there is a diminishing return on this. Serial peer review that does not materially change papers under consideration is a waste of everyone's time and expertise. At some point we need research where the ideas are stimulating debate. Such ideas are most likely to come from the original paper, not a version that has been revised time and again until no one can find areas of disagreement.

So do we need yet another journal for the fields of ecology and evolution? And if so, is it sufficient to simply add to the list or should it be different? Well, the growth and increasing importance of both fields suggests that the answer to the first question is yes, and the increasing pressure on journals suggests that the answer to the second is that there is an opportunity to do something different. *Ecology and Evolution* will be different. Our goal is to improve the efficiency of the process for everyone involved while maintaining the integrity that peer review delivers, and providing an outlet for the dissemination of important research. We will begin to make more efficient use of the excellent work done by reviewers and allow authors to promote their work, as closely as possible, in the form they originally intended. In doing so, this journal will publish first-rate papers across the spectrum of ecology, evolution and conservation.

My goal with *Ecology and Evolution* is to provide a journal where we are considering what the authors want while respecting what the reviewers require. Journals that are produced as paper copy as well as online have page limits, resulting in limits in the number and length of papers that are published. As an open access online-only journal, we are freed from this constraint. Shorter papers are read more often and cited more, and are encouraged, but we can allow authors the space to develop their ideas. More importantly, we can allow them to express ideas where not everyone agrees. One of my favourite sayings (I have many – you don't live in Kentucky for 8 years without picking up a few) is that I would rather be wrong than boring. We won't publish things that are wrong, but I also hope that we can avoid the continual polishing of research ideas until the shine of opinion is eliminated. In my ideal world, authors don't necessarily revise their manuscript exactly as proposed by the reviewers. However, they have to acknowledge the disagreement and should address it in their paper. Nothing is more frustrating to me as an editor than to get in a cover letter a perfectly argued response to a reviewer comment that finishes “so we did not change the manuscript”. Well, but what if other readers of the paper have the same concerns or misunderstandings? Better to force the discussion into the open. Educate others to your view. Have the discussion become a citable part of the literature.

*Ecology and Evolution* works with other journals in the Wiley portfolio, including journals that are owned by leading societies, to provide an opportunity for authors to transfer their work if it is not accepted by the original journal. You can see these partners on our homepage – quite a line-up. All of them have very high rejection rates and so there is the real opportunity to reduce reviewer overload. Where authors agree, their manuscript and the accompanying reviews are transferred and a rapid decision can be made. We also welcome direct submissions, which will undergo full peer review, and I intend to work to balance my author/editor hats and accept that both the reviewers and the authors can be right and that both views should be aired.

I believe that by making full use of the effort put into peer review across some of the highest profile journals in our fields we will maximize the contribution of the outstanding work done by authors and peer reviewers. *Ecology and Evolution* will, as a result, publish some of the very best papers we can produce while preserving the originality of thought and diversity of opinion that is such a vital part of research.

